# Supplementary material for: Bayesian refinement of protein structures and ensembles against SAXS data using molecular dynamics
Source: PLoS Comput Biol. 2017 Oct 18;13(10):e1005800. doi: 10.1371/journal.pcbi.1005800 (PMC5662244; doi:10.1371/journal.pcbi.1005800)
Supplement: S1 Archive — (BZ2) [file pcbi.1005800.s011.tar.bz2 › gromacs_4.62_bayesian_saxs_refi/share/html/online/g_sham.html]

g\_sham


| g\_sham Main Table of Contents |

**VERSION 4.6  
Sat 19 Jan 2013**

---

### Description

g\_sham makes multi-dimensional free-energy, enthalpy and entropy plots.
g\_sham reads one or more .xvg files and analyzes data sets.
The basic purpose of g\_sham is to plot Gibbs free energy landscapes
(option -ls)
by Bolzmann inverting multi-dimensional histograms (option -lp),
but it can also
make enthalpy (option -lsh) and entropy (option -lss)
plots. The histograms can be made for any quantities the user supplies.
A line in the input file may start with a time
(see option -time) and any number of y-values may follow.
Multiple sets can also be
read when they are separated by & (option -n),
in this case only one y-value is read from each line.
All lines starting with # and @ are skipped.

Option -ge can be used to supply a file with free energies
when the ensemble is not a Boltzmann ensemble, but needs to be biased
by this free energy. One free energy value is required for each
(multi-dimensional) data point in the -f input.

Option -ene can be used to supply a file with energies.
These energies are used as a weighting function in the single
histogram analysis method by Kumar et al. When temperatures
are supplied (as a second column in the file), an experimental
weighting scheme is applied. In addition the vales
are used for making enthalpy and entropy plots.

With option -dim, dimensions can be gives for distances.
When a distance is 2- or 3-dimensional, the circumference or surface
sampled by two particles increases with increasing distance.
Depending on what one would like to show, one can choose to correct
the histogram and free-energy for this volume effect.
The probability is normalized by r and r^2 for dimensions of 2 and 3,
respectively.
A value of -1 is used to indicate an angle in degrees between two
vectors: a sin(angle) normalization will be applied.
**Note** that for angles between vectors the inner-product or cosine
is the natural quantity to use, as it will produce bins of the same
volume.

### Files

| option | filename | type | description |
| --- | --- | --- | --- |
| **-f** | graph.xvg | Input | xvgr/xmgr file |
| **-ge** | gibbs.xvg | Input, Opt. | xvgr/xmgr file |
| **-ene** | esham.xvg | Input, Opt. | xvgr/xmgr file |
| **-dist** | ener.xvg | Output, Opt. | xvgr/xmgr file |
| **-histo** | edist.xvg | Output, Opt. | xvgr/xmgr file |
| **-bin** | bindex.ndx | Output, Opt. | Index file |
| **-lp** | prob.xpm | Output, Opt. | X PixMap compatible matrix file |
| **-ls** | gibbs.xpm | Output, Opt. | X PixMap compatible matrix file |
| **-lsh** | enthalpy.xpm | Output, Opt. | X PixMap compatible matrix file |
| **-lss** | entropy.xpm | Output, Opt. | X PixMap compatible matrix file |
| **-map** | map.xpm | Output, Opt. | X PixMap compatible matrix file |
| **-ls3** | gibbs3.pdb | Output, Opt. | Protein data bank file |
| **-mdata** | mapdata.xvg | Input, Opt. | xvgr/xmgr file |
| **-g** | shamlog.log | Output, Opt. | Log file |

### Other options

| option | type | default | description |
| --- | --- | --- | --- |
| **-[no]h** | bool | no | Print help info and quit || **-[no]version** | bool | no | Print version info and quit || **-nice** | int | 19 | Set the nicelevel || **-[no]w** | bool | no | View output .xvg, .xpm, .eps and .pdb files || **-xvg** | enum | xmgrace | xvg plot formatting: xmgrace, xmgr or none || **-[no]time** | bool | yes | Expect a time in the input || **-b** | real | -1 | First time to read from set || **-e** | real | -1 | Last time to read from set || **-ttol** | real | 0 | Tolerance on time in appropriate units (usually ps) || **-n** | int | 1 | Read this number of sets separated by lines containing only an ampersand || **-[no]d** | bool | no | Use the derivative || **-bw** | real | 0.1 | Binwidth for the distribution || **-[no]sham** | bool | yes | Turn off energy weighting even if energies are given || **-tsham** | real | 298.15 | Temperature for single histogram analysis || **-pmin** | real | 0 | Minimum probability. Anything lower than this will be set to zero || **-dim** | vector | 1 1 1 | Dimensions for distances, used for volume correction (max 3 values, dimensions > 3 will get the same value as the last) || **-ngrid** | vector | 32 32 32 | Number of bins for energy landscapes (max 3 values, dimensions > 3 will get the same value as the last) || **-xmin** | vector | 0 0 0 | Minimum for the axes in energy landscape (see above for > 3 dimensions) || **-xmax** | vector | 1 1 1 | Maximum for the axes in energy landscape (see above for > 3 dimensions) || **-pmax** | real | 0 | Maximum probability in output, default is calculate || **-gmax** | real | 0 | Maximum free energy in output, default is calculate || **-emin** | real | 0 | Minimum enthalpy in output, default is calculate || **-emax** | real | 0 | Maximum enthalpy in output, default is calculate || **-nlevels** | int | 25 | Number of levels for energy landscape || **-mname** | string |  | Legend label for the custom landscape |

---

http://www.gromacs.org  
gromacs@gromacs.org
